# Global Research Output on Sleep Research in Athletes from 1966 to 2019: A Bibliometric Analysis

**DOI:** 10.3390/clockssleep2020010

**Published:** 2020-03-30

**Authors:** Michele Lastella, Aamir Raoof Memon, Grace E. Vincent

**Affiliations:** 1 Appleton Institute of Behavioural Science, Central Queensland University, Adelaide 5034, South Australia, Australia; m.lastella@cqu.edu.au (M.L.); g.vincent@cqu.edu.au (G.E.V.); 2 Institute of Physiotherapy and Rehabilitation Sciences, Peoples University of Medical and Health Sciences for Women, Nawabshah (SBA), Sindh, Pakistan

**Keywords:** athletic, bibliometrics, research output, scientific production, sports, sleep

## Abstract

This study examined sleep research in athletes published between 1966 and 2019, through a bibliometric analysis of research output in the Scopus database. Following a robust assessment of titles, the bibliometric indicators of productivity for studies included in the final analysis were: Distribution of publications and citations (excluding self-citations), top ten active journals, countries, institutions and authors, single- and multi-country collaboration, and 25 top-cited papers. Out of the 1015 papers, 313 were included in the final analysis. The majority of the papers were research articles (*n* = 259; 82.8%) and published in English (*n* = 295; 94.3%). From 2011, there was a dramatic increase in papers published (*n* = 257; 82.1%) and citations (*n* = 3538; 91.0%). The number of collaborations increased after 2001, with papers published through international (*n* = 81; 25.9%) and national (*n* = 192; 61.3%) collaboration. Australia was the most prolific country in terms of number of publications (*n* = 97; 31.0%), and citations (*n* = 1529; 15.8%). In conclusion, after the beginning of the twenty-first century, the scientific production on sleep research in athletes has seen significant growth in publication and citation output. Future research should focus on interventions to improve sleep in athletes.

## 1. Introduction

Sleep is a basic requirement for human health and serves critical psychological and physiological functions [1,2]. To maintain optimal health and functioning, adults should obtain at least 7 h of sleep per night [3]. Epidemiological and experimental studies have shown that not obtaining adequate sleep is linked to a number of adverse psychological and physiological outcomes, including impairments to cognitive performance [4,5], mood [6], appetite regulation [7], as well as critical metabolic [8], and immunologic processes [9]. Sleep has long been identified by athletes and coaches as a critical component for training and competition. In recent years, there has been a dramatic increase in studies investigating the relationship between sleep, recovery, and athletic performance [10,11]. For athletes, frequent exposure to high training loads and competition demands increases recovery needs, and therefore, may increase their overall sleep requirement [10]. The first study to investigate relationships between sleep, recovery and performance in athletes was conducted by Baekeland et al. [12]. The authors noted increases in Slow Wave Sleep (SWS) following strenuous afternoon exercise. However, the next seminal investigation was not until 30 years later where Taylor et al. [13], noted increases in SWS during an athletic peak competitive phase. This study initiated a substantial increase in studies examining the impact of sleep in athletes, including relationships with training [14], competition [15,16], injury [17], psychological well-being [18], and performance [19]. However, to date, this growth in research has not been captured empirically. 

Bibliometric techniques aim to understand the research trends and focus, and contributions to scholarship by any field, country, institution, author or a journal [20,21]. The outcomes of these techniques are important for funding agencies, as they provide objective information about the quantity and quality of the research activity [22,23,24,25]. Further, examining author keywords and/or content analysis reveals current and future trends within a specific field [22,23,26,27]. The utilisation of bibliometric analyses are becoming increasingly common across a number of fields, including physiotherapy, human trafficking, road traffic injuries, m-health, nursing and midwifery and physical activity and sleep [27,28,29,30,31,32,33,34]. Previous studies have attempted to catalogue the evolution of sleep research, sleep research in medicine and biology, and trends in sleep research through bibliometric techniques, but the existing evidence is more than a decade old [35,36,37]. In addition, a bibliometric analysis of sleep research in athletes has not been conducted to date. Therefore, the aim of this study was to conduct a bibliometric analysis of sleep research in athletes. Although different databases, such as Web of Science, PubMed/Medline and SPORTDiscus may be used to achieve this, Scopus was used in the current study because of its advantages over other bibliographic databases [38,39,40]. The findings of this study will provide an in-depth insight into the current state of the literature and trends in the area of sleep in athletes and provide directions for future research.

## 2. Materials and Methods

### 2.1. Database and Search Strategy

This study does not involve human subjects, and therefore, did not require ethics approval. Scopus (https://www.scopus.com/) was used because it is a multidisciplinary bibliographic database, covering over 23000 peer-reviewed journals, and is one of the largest research databases [40]. Scopus is also commonly used in bibliometric studies [27,31,32,33,34,41]. Bibliometric analyses differ from the methodology employed in systematic reviews; the discussion about the typology of reviews could be found elsewhere [20,41,42,43]. The use of title search is common in bibliometric studies in contrast to the title-abstract-keywords search query [32,33,34,41]. The search strategy for the current study was informed by the methodology used in previous bibliometric analyses [28,31,34,41]. 

Use of appropriate and relevant keywords is essential for improving the accuracy of the findings, and it directly affects the findings of a bibliometric study [33]. In order to determine relevant keywords, several recent systematic reviews on sleep in athletes were examined [10,44,45,46]. The literature search was performed following several steps to ensure that the retrieved results were obtained with maximum accuracy, limiting the likelihood of false positive results. First, an online search of the literature on sleep in athletes was conducted via the Scopus database on August 14, 2019. The results of the initial search strategy were assessed by all the authors to ensure that all relevant keywords were included and the first paper on sleep in athletes was identified; it was decided that the retrieved literature will cover the period from 1965 to 2019 because the first paper about sleep in athletes was published in 1966 [12]. Second, the inclusion and exclusion criteria were then refined to reduce the likelihood of false positive results. Finally, a randomly selected list of titles was assessed twice, and after an extensive discussion, a final search query was run on August 21, 2019. Eighty general and specific keywords for searching the literature on sleep in athletes were used, which included “sleep*”, “sleep quantity”, “sleep quality”, “sleep-hygiene”, “sleep disturbance”, “nap*”, “wak*”, “wake”, “athlet*”, “player*”, “sport*”, “competit*”, “team sport*”, “individual sport*”, “game*”, “cyclist*”, “football*”, “basketball*”, “netball*”, “cricket*”, “handball*”, “baseball*”, “softball*”, “rugby”, “cycling”, “soccer”, “*hockey”, “futsal”, “volleyball”, “korfball”, “lacrosse”, “curling”, “polo”, “*swimming”, “rowing”, “mountain biking”, “triathlon”, “race walking”, “canoeing”, “diving”, “running”, “judo”, “*skating”, “ballet”, “skiing”, “snowboarding”, “taekwondo”, “archery”, “badminton”, “bmx”, “bocce”, “bowling”, “boxing”, “cheerleading”, “croquet”, “dance”, “fencing”, “golf”, “gymnastics”, “martial arts”, “squash”, “throw”, “*tennis”, “karate”, “touch football*”, “ice-hockey”, “synchronised swimming”, “swimming”, “speed skating”, “figure skating”, “table tennis”, “weight lift*”, “roller skating”, “motor sport*”, “orienteering”, “equestrian”, “wheelchair prop”, “water polo”, “abseiling”, and “surfing”. The keywords were combined using Boolean operators (AND and OR), and wild cards/truncations were used with some keywords to look for their word variants (Appendix A).

### 2.2. Inclusion and Exclusion Criteria

Studies exploring sleep in athletes were included. An athlete was defined as someone who “engages in physical activity or sports with the primary goal of improving performance to bolster athletic excellence and/or achievement” [47,48,49]. This included ‘recreational’, ‘retired’, ‘semi-elite’, ‘competitive elite’, ‘successful elite’ and ‘world-class elite’ athletes [47,48,49]. Recreational athletes were defined as “recreational participants who are adequately trained and adequately skilled to be categorised as athletes” [47]. Retired athletes referred to the athletes who took retirement from their sport career. Swann et al. provide the definition of semi-elite, competitive elite, successful elite and world-class elite athletes [49]. Studies that investigated clinical sleep disorders in athletes (such as insomnia, rapid eye movement (REM) sleep behaviour disorder, parasomnias, circadian rhythm disorders, sleep-related movement disorders, etc.) were included. Furthermore, studies investigating the relationship between sleep and health outcomes (e.g., depression, body temperature, etc.) in athletes were included. 

The exclusion criteria were: (1) Studies on non-athletic populations (e.g., healthy sedentary or physically active participants), industrial or occupational athletes (e.g., military personnel, firefighters) were excluded; (2) studies validating a tool/device or methodological studies (i.e., accelerometer algorithms for sleep, psychometric properties of questionnaires, etc.) in athletes; (3) studies conducted on animals; and (4) studies published in languages other than English lacking an English abstract. In the case of papers published in English but without an abstract, abstracts were manually searched to determine the inclusion of the paper. 

Only papers published in journals were included, whereas, books, book chapters, and conference proceedings were excluded from analyses. Errata papers were also excluded from the analyses, but conference papers were included (as they cannot be published twice). To encompass all the relevant literature, irrespective of the language of publication, in this bibliometric analysis, no restrictions were made for the language of the published papers.

### 2.3. Data extraction, Validation and Analysis (Bibliometric Indicators and Mapping)

**Figure 1:**
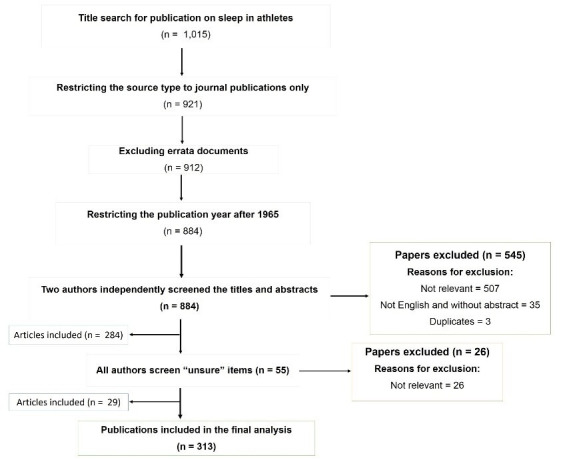
Flow diagram for title selection for bibliometric analysis.

Two authors (ML and GV) independently scored 25 randomly selected titles and abstracts for inclusion or exclusion. This was done three times to validate the search strategy and ensure the appropriateness of the inclusion and exclusion criteria. Following this, two authors (ML and GV) independently reviewed the titles and abstracts of all the papers (*n* = 884) and irrelevant papers (i.e., not meeting the inclusion/exclusion criteria) (*n* = 507), papers not published in English that are without abstract (*n* = 35) and duplicate papers (*n* = 3) were excluded. Fifty-five papers, where the two authors (ML and GV) were unsure, were discussed among all authors (ML, GV, and ARM) and irrelevant papers (26 out of 55) were excluded. Finally, 313 (35.4%) papers were deemed eligible for inclusion in the final analysis (Figure 1; supplementary file). 

The data for retrieved papers were imported directly from the Scopus database as a CSV (comma-separated values) file and analysed using Microsoft Excel 2013 (Microsoft Corporation, Santa Rosa, California, USA), SPSS v20, and VOSViewer program. The study findings were presented through graphs, tables and network visualisation maps. The following information for the papers was included in the final analysis: The type of the papers, the total number of papers, total number of citations to the retrieved papers (excluding self-citations), 10 most active journals, countries, institutions and authors, and 25 top-cited papers. Growth in the number of publications was reported through stratified growth rate (SGR) across six strata of nine years period, including period-1 (1966–1974), period-2 (1975–1983), period-3 (1984–1992), period-4 (1993–2001), period-5 (2002–2010) and period-6 (2011–2019). The SGR was a modification of the annual growth rate (AGR), and was based on the equation: (Ending Value − Beginning Value)/Beginning Value] × 100 [34]. After excluding the self-citations, the number of citations and citations per paper were calculated and reported across these six time periods. Authorship pattern was determined by calculating number of single- and multi-authored papers, the total number of authors and number of authors in multi-authored papers, the average number of authors per paper. The collaboration was ascertained by calculating papers with single-country (national) and multi-country (international) collaboration. Single authored papers were also reported. In addition, author keywords were used to present the emerging trends and focus of the sleep research in athletes. The journal impacts factor (JIF), SCImago Journal Rank (SJR), and CiteScore were reported, for the leading journals publishing sleep research in athletes, as they are commonly used measures of scientific influence of scholarly journals [50,51]. The data for JIF was obtained from the Journal Citation Report (JCR) for the year 2018, SJR (https://www.scimagojr.com/) and CiteScore (https://journalmetrics.scopus.com/) from Elsevier. The Hirsch index (h-index) for the leading journals, authors, institutions, and countries was also reported as a proxy measure of the influence of an author, country or an institution [52,53]. 

For the top-cited papers, 25 papers for sleep research in athletes and their citation, the Scopus were presented. As the total number of citations has several limitations [54,55,56], citation density (citations per year) was used, in the current paper, as a measure to determine the impact of a research paper among the top-cited papers. Citation density (often called citation rate or citations per year) was calculated by dividing a paper’s citation count (total citations) by the number of years since publication [54]. Instead of conducting a separate analysis and present it elsewhere, the cutoff of 25 papers was selected in the current study to show the most impactful papers on sleep in athletes. 

The Java program VOSviewer, a freely available software by Leiden University Netherlands, was used for graphic presentation of co-authorship analysis, the co-occurrence of author keywords, and international collaboration among countries through network visualisation maps. This program is widely used to map and visualise the citation relationships [57,58]. The features of network visualisation maps are reflected through specific colours (clusters) representing units (i.e., keywords) belonging to one group, size of the circle and font size proportional to the productivity, occurrence, number of citations, and thickness of connecting lines indicating the strength of collaboration. 

## 3. Results

### 3.1. Type of Documents and Growth Pattern

**Figure 2:**
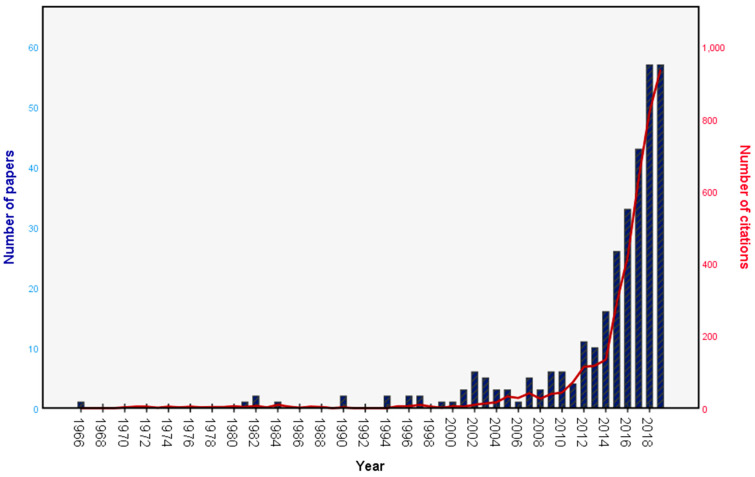
The growth trend for publications and citations (1966–2019).

In total, 313 (35.4%) documents were included in the final analysis. The highest number of papers by type were research articles (*n* = 259; 82.8), reviews (27; 8.6%) and letters (13; 4.2%) (Table 1). All the retrieved papers were published in eight languages, with English (295; 94.3%) as the most common language. Although a number of publications and citations started to grow after the early 2000s, there was a sharp increase in the number of citations after 2007, but the publication output rapidly grew only after 2011 (Figure 2). A total of 1,542 authors (ranging from 1 to 18 authors per paper) participated in publishing sleep research in athletes, with an average of 4.9 authors per paper. The national collaboration was present in 203 (64.9%) papers and international in 81 (25.9%) papers. In addition, 25 (8%) papers were single-authored, and 288 (92%) were multi-authored. 

There was only one paper published during the period from 1966 to 1974. The initial 27 years of the analysis showed output fluctuation, but after 1992, there was a continuous rise in the number of authors, average authors per paper, single-authored papers, multiple-authored papers and single country (national) collaboration (Table 2). However, multi-country (international) collaboration increased after 2001, with 81 (25.9%) published papers published by authors through international collaboration. The period from 2011 onwards was very productive, with 257 (82.1%) papers and 3538 (91.0%) citations—a growth by 576.3%. There were 57 (18.2%) papers published in 2019 which, based on previous output, is predicted to rise to approximately 86 published papers by the end of the year.

### 3.2. Preferred Journals and Highly Cited Papers

In total, 121 journals published the included papers, and top 10 journals published 126 (40.3%) of the total number of papers. *European Journal of Sport Science* and *Journal of Sports Sciences* were ranked first with 17 (5.4%) papers each, followed by *International Journal of Sports Physiology and Performance* (16; 5.1%) and *Chronobiology International* (15; 4.8%). In addition, h-index of the top 10 journals was in the range of 4 to 9. Five of the top ten journals were from the UK, three from the USA, and one from the Netherlands and Italy (Table 3). There were only nine papers published in *Sports Medicine*, which received 355 citations (citation per paper = 39.4)—the highest amongst the top 10 journals. All the journals had the journal impact factor and CiteScore above one (with *Biological Rhythm Research* being one exception). *British Journal of Sports Medicine* had the highest impact factor (11.645; 5-year IF = 9.805) and published 12 (3.8%) papers. Seven journals belonged to Q1, two to Q2 (SJR: 0.54–0.98) and one was from Q3 (SJR = 0.28).

From the citation analysis of 25 highly-cited papers, it was found that eighteen papers were research articles, five reviews, and two conference papers. The national collaboration was present in sixteen papers and international collaboration in six papers. Two papers were single-authored (Table 4). The highest number of citations were received by the paper titled “*The effects of sleep extension on the athletic performance of collegiate basketball players*” [19], which was published in *Sleep* in 2011, and received a total of 158 citations according to Scopus. On the other hand, the second-ranked paper was a review titled “*Sleep and athletic performance: The effects of sleep loss on exercise performance, and physiological and cognitive responses to exercise*” [59], with the highest citation density (29.2 citations per year since publication) and was published in 2015. The paper “*Sleep in elite athletes and nutritional interventions to enhance sleep*” [60] was a single-authored research article, among the top 10 highly cited papers, with 104 citations. The first paper on sleep in athletes titled “*Exercise and sleep patterns in college athletes*” [12], published in *Perceptual and Motor Skills* in 1966, was ranked 8th and had received only 103 citations (1.9 citations per year since publication). Fifteen (60%) of the top 25 highly cited papers were published after 2010. 

### 3.3. Highly Productive Countries, Institutions and Pattern of Collaboration

Authors from 39 countries and 160 affiliations participated in publishing sleep research in athletes. The top 10 countries and institutions participated in publishing 311 (99.4%) and 153 (48.9%) papers, respectively. Australia was the most productive country with 97 (31.0%) publications, 1529 (39.4%) citations, and 49 (15.7%) national and 43 (13.7%) international collaborative papers, with an h-index of 21. A total of 520 authors from Australia contributed to publishing the papers included in this bibliometric study (Table 5). With 66 (21.1%) papers, the United States was ranked second, followed by France (25; 8.0%) and the United Kingdom (24; 7.7%). Four countries in the list of top 10 were from Europe, two from Asia, two from Western Pacific, one from North America, one from Latin America. Tunisia was the most productive African country with 11 (3.5%) papers and stood at 12th in the list of highly productive countries (data not presented). Germany, with the value of 22.1, had the highest citations per paper and stood at 5th position with 20 (6.4%) publications and 442 citations. The highest number of authors per paper was recorded for Brazil, with an average of 7.6 authors per paper, contributing to only 15 (4.8%) publications.

**Figure A1:**
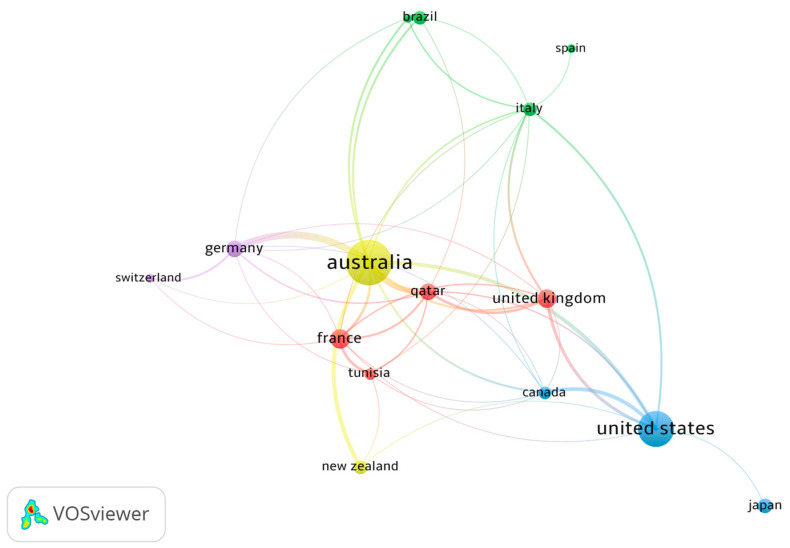
Network visualisation map for countries with minimum five papers.

**Figure A2:**
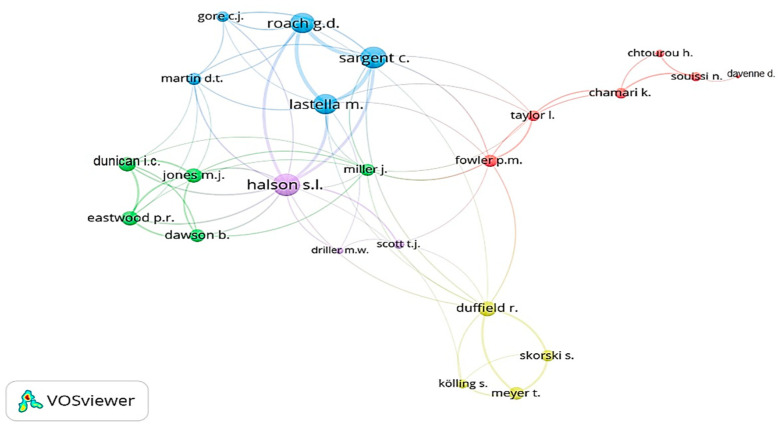
Network visualisation map for authors with minimum five papers.

The network visualisation map for countries with minimum of five papers consisted of 15 countries in five clusters (Figure A1). The strongest collaboration was present among Australia–Germany (link strength = 13), Australia–Qatar (link strength = 10), and Australia–New Zealand (link strength = 7). Co-authorship network analysis for authors with at least five papers is shown in the supplementary material (Figure A2). 

The top 10 most productive institutions were from Australia (*n* = 5), Brazil (*n* = 1), Germany (*n* = 1), New Zealand (*n* = 1), Qatar (*n* = 1), and the UK (*n* = 1). The Australian Institute of Sport (Australia) was ranked first and contributed to 47 (15.0%) published papers, with 760 citations (h-index = 16). The institutions ranked at the second, third and fourth position were *Central Queensland University* (Australia), *University of Technology Sydney* (Australia) and *University of Western Australia* (Australia), respectively (Table 6). *Universität des Saarlandes* (Germany) had the highest citations per paper 30.4 and received 274 citations for 9 (2.9%) published papers.

### 3.4. Prolific Authors and Authorship Pattern

One-hundred and sixty authors published 313 papers. Eight authors in the list of ten most prolific authors were from Australia, one from Germany and one from Qatar (Table 7). With 25 (8.0%) published papers and 431 citations (17.2 citations per paper; h-index = 9), *Shona L. Halson* of *Australian Catholic University* (Australia) was the most productive author. The next three authors from *Central Queensland University* (Australia) were *Charli Sargent* with 20 (6.4%) publications and 286 citations (14.3 citations per paper; h-index = 9), *Michele Lastella* with 16 (5.11%) publications and 216 citations (13.5 citations per paper; h-index = 5) and *Gregory D. Roach* with 16 (5.1%) publications and 221 citations (13.8 citations per paper; h-index = 7). However, *Tim F. Meyer* of *Universität des Saarlandes, Saarbrucken* (Germany), with 9 (2.9%) publications and 274 citations, had the highest citations per paper (30.4). *Michele Lastella* had the highest number of papers (9 out of 16) published as the first author, whereas, *Charli Sargent* has the highest number of papers (11 out of 20) published as a senior/last author. 

### 3.5. Network Visualisation Map (Collaborative Networks and Research Trends)

**Figure 3:**
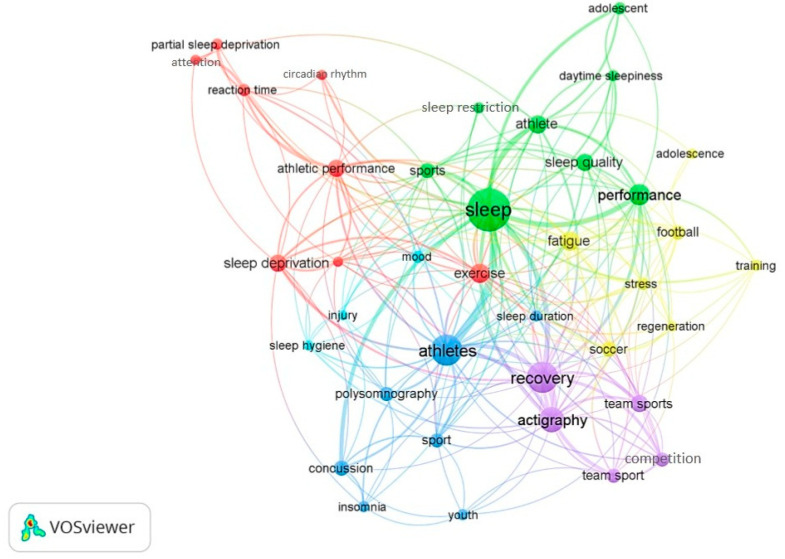
Network visualisation map for author keywords with a minimum occurrence of 5.

The network visualisation map yielded 6 clusters of 38 keywords for author keywords with minimum occurrences of 5. There were a total of 225 links, which indicates links of an item with other items, with a total link strength (total strength of the links of an item with other items) of 443 (minimum link strength = 3, maximum link strength = 112) and occurrence ranging from 5 to 76. This suggests that there were 225 pairs of 38 keywords having the co-occurrence links. In simple words, sleep quality and athlete co-occur together making a link of co-occurrence. This link has a link strength, represented by the number of documents in which two keywords occur together. The sum of the link strengths, reflective the pattern in which group of keywords co-occur, was 443. The top 15 most common author keywords were sleep, athletes, recovery, actigraphy, performance, fatigue, exercise, athlete, sleep deprivation, sleep quality, athletic performance, team sports, sports, concussion, and soccer (Figure 3). There were eight keywords each in cluster 1 and 2, seven keywords in cluster 3 and 4, five keywords in cluster 5, and three keywords in cluster 6. Keywords in the same cluster are represented by the same colour and usually co-occur together. For instance, the yellow-coloured cluster shows that adolescence, fatigue, football, regeneration, soccer, stress, training are commonly listed together.

## 4. Discussion

This study provides a comprehensive bibliometric analysis of the published scientific literature examining sleep research in athletes. Over the last decade (2010–2019) there has been substantial growth in research pertaining to sleep in athletes, with 257 new research articles published. Similar to sports nutrition research [61], sleep research in athletes has progressed, due to the well-established importance of sleep toward optimising recovery and performance [59,62]. This trend may reflect the growing interests of athletic/sporting organisations and the scientific community in understanding the critical role sleep has on athlete health, recovery and performance. Technological advancements in sleep measurement, such as the implementation of research-grade accelerometers in athlete populations may have also contributed to the rise in research output [63]. 

The increased production of sleep research in athletes in the past decade (2010–2019) was led by Australia, closely followed by the United States of America, France, the United Kingdom, and Germany. This increased production, mostly in developed countries, may reflect the globalisation of sport and the large investments made by governing bodies [64,65]. For example, in the 2016–2017 financial year, the Australian government committed $101 million (AUD) to high performance sports funding [66]. For many countries, investment into sport is viewed in light of a virtuous cycle such that elite sporting success offers international prestige for the nation, a certain ‘feel-good factor among the people’ and ultimately can increase sport participation throughout the population [65]. Further, investment into sports specific scientific research and employment of sports scientists and specialist consultants (e.g., dedicated sports scientists) is becoming more prevalent and contributes to improving sporting performance [67]. 

In this study, literature investigating sleep in athletes was dominated by multi-authored articles, articles written in English and original investigations. Previous bibliometric studies indicate that original articles and papers are written in English typically make up the majority of the assessed literature [27,32,33,34]. In the current study, 40% of the publications (average 14 citations per publication) were from the top ten journals, which had an average impact factor of 4.0. Further, 70% of the top ten journals were from the list of top 25% (i.e., Q1) of journals for at least one of their classified sub-disciplines. According to the article type classification in the Scopus database, five reviews were included in the top 25 highly cited papers covering topics, such as sleep, recovery, circadian rhythms, cognitive and athletic performance. From these metrics, it is evident that sleep research in athletes has predominantly focused on a) monitoring athletes sleep during training and competition [15,16,68,69], b) the physiological and psychological demands of different sports on sleep [70,71,72], c) examining factors, such as travel [73], altitude [74,75], training load [71], and competition anxiety [16,76,77] that either impact athletes’ ability to sleep or d) how psychological well-being [18,78], and recovery [71] are influenced by sleep. The keyword visualisation map illustrates the bi-directional relationships between sleep, recovery and performance (Figure 3). Data derived from the highest cited papers and visualisation map indicate that most research is based on travel, the timing of training/competition, the influence of training load on sleep and injury [15,16,17,71,73]. Further, the importance of sleep before and after sport-related concussions has also become a topic of growing interest [79,80].

This bibliometric analysis has highlighted areas within the field of sleep and athletes that require further research. Of note, the highest cited paper by Mah et al. showed that extending sleep duration by 1.8 h per night improved college athletes’ overall athletic performance [19]. However, since this publication, there have only been two studies that have found improvements in sport-specific performance following sleep extension [81,82]. In contrast, Famodu et al. observed no differences in performance following sleep extension [83]. Given the significant impact of Mah et al. in terms of citation count [19], it is evident that future investigations need to implement sleep extension protocols to ensure the recommendations of extending sleep duration are based on sound empirical evidence. 

Another strategy to improve sleep duration in athletes that have been largely understudied with the sleep research in athletes is napping. For an athlete, a daytime nap is often employed to counteract sleep debt, increase sleep duration over a 24 h period and reduce daytime sleepiness [84]. Given the growth sleep research in athletes has received over the past decade, it seems negligent that few studies have included nap duration as part of an athletes’ sleep over a 24 h period [14,19]. Moving forward, it is imperative that studies collect data on the timing and duration of naps taken by athletes at a range of different times (e.g., pre- and post-competition, during an intensive training period). In doing so, total sleep duration over a 24 h period can be obtained, rather than just the main night time sleep periods. Therefore, future studies should consider examining the benefits and risks of napping in relation to athlete recovery and performance. Other areas of future research include the bi-directional relationships between sleep and injury, as well as the impact of sports related concussion on subsequent sleep. 

The main strengths of the current study comprising of robust procedures were: Rigorous selection criteria and assessment of titles-abstracts and full-text (in some cases) for the suitability, the inclusion of journal publications without restricting the language of publication, and presentation of citation impact through h-index after limiting author’ self-citations and citation density for top-cited papers. However, the current study has some limitations, which should be acknowledged. Although we aimed to include all the relevant journal publications on sleep research in athletes available in the Scopus database, non-indexed journals and publications might not have been recognised. In addition, most of the journals indexed in the scholarly databases like Scopus are published in English, which suggests that the papers published in non-English journals and in journals not indexed in the Scopus database were, therefore, not included in the study [27,85]. Secondly, the bibliometric indicators presented in the current study were based on the data directly imported from the Scopus database and in some cases, there might be inaccuracies in the names of authors, institutions, and in the categorisation of papers by document type—an inherent limitation of bibliographic databases [27,85]. Finally, the title search is commonly used in bibliometric studies and is expected to yield accurate data, thereby reducing the potential for false-positive and false-negative results. However, this strategy might miss the publications where keywords are mentioned in the title of the paper. We tried to limit the inclusion of false-positive by manually reviewing the title and abstract of each paper, but we could not limit the likelihood of false-negatives. It should be noted that these limitations are common to bibliometric studies [27,32,33,34,41].

## 5. Conclusions

This is the first study to examine the bibliometric parameters of sleep research in athletes since the first published study in 1966. The present study showed the sleep research in athletes has grown considerably over the last decade (2010–2019). The increasing number of citations over and a relatively high h-index of the retrieved papers indicates the importance of sleep for athletes, sports practitioners, researchers, clinicians and policy makers. Future research in this field should go beyond sleep monitoring, and focus on interventions (e.g., sleep extension, napping) to improve the sleep of athletes.

## Figures and Tables

**Table 1:** Type of papers on sleep research in athletes.

Type of Paper	Frequency (*n* = 313)	Percentage (%)
Article (including articles in press)	259	82.8
Review	27	8.6
Letter	13	4.2
Conference Paper	5	1.6
Note	4	1.3
Short Survey	3	1.0
Editorial	2	0.6

**Table 2:** Growth pattern for sleep research in athletes.

Year Group	Papers	Cumulative Papers	SGR	Citations (min-max)	Cumulative Citations	CPP	Authors	AAPP	SAP	MAP	AMAP	AAMAP (min-max)	SCC	MCC *
1966–1974	1	-	-	15 (1–4)	-	15.0	2	2.0	0	1	2	2.0 (2–2)	0	0
1975–1983	3	4	200	30 (2–6)	45	10.0	4	1.3	2	1	2	2.0 (2–2)	0	0
1984–1992	3	7	0	23 (0–9)	68	7.7	13	4.3	1	2	12	6.0 (6–6)	2	0
1993–2001	11	18	266.7	33 (0–9)	101	3.0	42	3.8	2	9	40	4.4 (2–6)	9	0
2002–2010	38	56	245.5	247 (9–43)	348	6.5	184	4.8	4	34	180	5.3 (2–13)	27	7
2011–2019	257	313	576.3	3538 (72–937)	3886	13.8	1297	5.1	16	241	1281	5.3 (2–18)	165	74
Total	313	313	-	3886 (0–937)	3886	12.4	1542	4.9	25	288	1517	5.3 (2–18)	203	81

SGR = stratified growth rate; CPP = citation per paper; APP = average authors per paper; SAP = single authored paper; MAP = multi authored paper; AMAP = authors in multi authored paper; AAMAP = average authors in multi authored paper; SCC = single country collaboration; MCC = multi-country collaboration. * undetermined in 4 papers.

**Table 3:** Top 10 preferred journals for sleep research in athletes (*n* = 313).

Journal	Country	Papers (%)	Citations	CPP	h-index	JIF_2018_	5-year IF	SJR_2018_	CiteScore
*European Journal of Sport Science*	UK	17 (5.4)	239	14.1	9	2.376	2.896	1.17 (Q1)	2.75
*Journal of Sports Sciences*	UK	17 (5.4)	316	18.6	8	2.811	3.264	1.23 (Q1)	2.79
*International Journal of Sports Physiology and Performance*	USA	16 (5.1)	155	9.7	7	3.979	4.133	1.94 (Q1)	3.44
*Chronobiology International*	USA	15 (4.8)	189	12.6	6	2.562	2.998	0.98 (Q2)	2.61
*Biological Rhythm Research*	UK	13 (4.2)	85	6.5	4	0.773	0.691	0.28 (Q3)	0.85
*Journal of Science and Medicine in Sport*	Netherlands	13 (4.2)	217	16.7	7	3.623	4.198	1.67 (Q1)	3.84
*British Journal of Sports Medicine*	UK	12 (3.8)	132	11.0	7	11.645	9.805	4.14 (Q1)	6.02
*Sports Medicine*	UK	9 (2.9)	355	39.4	6	7.583	9.257	3.19 (Q1)	7.42
*Journal of Sports Medicine and Physical Fitness*	Italy	7 (2.2)	42	6.0	4	1.302	1.395	0.54 (Q2)	1.16
*Journal of Strength and Conditioning Research*	USA	7 (2.2)	44	6.3	4	3.017	3.101	1.5 (Q1)	2.59

CPP = citations per paper; JIF**_2018_** = Journal impact factor for 2018; 5-year IF = 5-year impact factor; SJR**_2018_** = SCImago Journal Rank for 2018.

**Table 4:** Top 25 highly-cited papers for sleep research in athletes.

R	Citation of the Paper	Collaboration	Pub. Type	Citations	CD
1	Mah, C. D., Mah, K. E., Kezirian, E. J., & Dement, W. C. (2011). The effects of sleep extension on the athletic performance of collegiate basketball players. *Sleep*, 34(7), 943–950.	SCC	Article	158	17.6
2	Fullagar, H. H., Skorski, S., Duffield, R., Hammes, D., Coutts, A. J., & Meyer, T. (2015). Sleep and athletic performance: The effects of sleep loss on exercise performance, and physiological and cognitive responses to exercise. *Sports Medicine*, 45(2), 161–186.	MCC	Review	146	29.2
3	Reilly, T., & Edwards, B. (2007). Altered sleep–wake cycles and physical performance in athletes. *Physiology & Behavior*, 90(2-3), 274–284.	SCC	Article	135	10.4
4	Leeder, J., Glaister, M., Pizzoferro, K., Dawson, J., & Pedlar, C. (2012). Sleep duration and quality in elite athletes measured using wristwatch actigraphy. *Journal of Sports Sciences*, 30(6), 541–545.	SCC	Article	119	14.9
5	Brand, S., Gerber, M., Beck, J., Hatzinger, M., Pühse, U., & Holsboer-Trachsler, E. (2010). High exercise levels are related to favourable sleep patterns and psychological functioning in adolescents: A comparison of athletes and controls. *Journal of Adolescent Health*, 46(2), 133–141.	SCC	Article	118	11.8
6	Ashenden, M. J., Gore, C. J., Dobson, G. P., & Hahn, A. G. (1999). “Live high, train low” does not change the total haemoglobin mass of male endurance athletes sleeping at a simulated altitude of 3000 m for 23 nights. *European Journal of Applied Physiology and Occupational Physiology*, 80(5), 479–484.	SCC	Article	106	5.1
7	Halson, S. L. (2014). Sleep in elite athletes and nutritional interventions to enhance sleep. *Sports Medicine*, 44(1), 13–23.	SAP	Article	104	17.3
8	Baekeland F., & Lasky R. (1966). Exercise and sleep patterns in college athletes. *Perceptual and Motor Skills*, 23(3), 1203–1207.	*	Article	103	1.9
9	Juliff, L. E., Halson, S. L., & Peiffer, J. J. (2015). Understanding sleep disturbance in athletes prior to important competitions. *Journal of Science and Medicine in Sport*, 18(1), 13–18.	SCC	Article	86	17.2
10	Gosselin, N., Lassonde, M., Petit, D., Leclerc, S., Mongrain, V., Collie, A., & Montplaisir, J. (2009). Sleep following sport-related concussions. *Sleep Medicine*, 10(1), 35–46.	MCC	Article	86	7.8
11	Milewski, M. D., Skaggs, D. L., Bishop, G. A., Pace, J. L., Ibrahim, D. A., Wren, T. A., & Barzdukas, A. (2014). Chronic lack of sleep is associated with increased sports injuries in adolescent athletes. *Journal of Pediatric Orthopaedics*, 34(2), 129–133.	SCC	Article	84	14
12	Samuels C. (2008). Sleep, recovery, and performance: The new frontier in high-performance athletics. *Neurologic Clinics*, 26(1), 169–180.	SAP	Review	84	7
13	Hynynen, E. S. A., Uusitalo, A., Konttinen, N., & Rusko, H. (2006). Heart rate variability during night sleep and after awakening in overtrained athletes. *Medicine and Science in Sports and Exercise*, 38(2), 313–317.	SCC	Article	81	5.8
14	Sargent, C., Lastella, M., Halson, S. L., & Roach, G. D. (2014). The impact of training schedules on the sleep and fatigue of elite athletes. *Chronobiology International*, 31(10), 1160–1168.	SCC	Conference Paper	78	13
15	Hausswirth, C., Louis, J., Aubry, A., Bonnet, G., Duffield, R., & LE Meur, Y. (2014). Evidence of disturbed sleep and increased illness in overreached endurance athletes. *Medicine and Science in Sports and Exercise*, 46(5), 1036–1045.	MCC	Article	75	12.5
16	Lastella, M., Roach, G. D., Halson, S. L., & Sargent, C. (2015). Sleep/wake behaviours of elite athletes from individual and team sports. *European Journal of Sport Science*, 15(2), 94–100.	SCC	Article	71	14.2
17	Reilly, T., & Piercy, M. (1994). The effect of partial sleep deprivation on weight-lifting performance. *Ergonomics*, 37(1), 107–115.	SCC	Article	68	2.6
18	Erlacher, D., Ehrlenspiel, F., Adegbesan, O. A., & Galal El-Din, H. (2011). Sleep habits in German athletes before important competitions or games. *Journal of Sports Sciences*, 29(8), 859–866.	MCC	Article	67	7.4
19	Nédélec, M., Halson, S., Abaidia, A. E., Ahmaidi, S., & Dupont, G. (2015). Stress, sleep and recovery in elite soccer: A critical review of the literature. *Sports Medicine*, 45(10), 1387–1400.	MCC	Review	62	12.4
20	Thun, E., Bjorvatn, B., Flo, E., Harris, A., & Pallesen, S. (2015). Sleep, circadian rhythms, and athletic performance. *Sleep Medicine Reviews*, 23, 1–9.	SCC	Review	57	11.4
21	Fietze, I., Strauch, J., Holzhausen, M., Glos, M., Theobald, C., Lehnkering, H., & Penzel, T. (2009). Sleep quality in professional ballet dancers. *Chronobiology International*, 26(6), 1249–1262.	SCC	Article	54	4.9
22	Lastella, M., Lovell, G. P., & Sargent, C. (2014). Athletes’ precompetitive sleep behaviour and its relationship with subsequent precompetitive mood and performance. *European Journal of Sport Science*, 14(sup1), S123–S130.	SCC	Article	51	8.5
23	McClure D.J., Zuckerman S.L., Kutscher S.J., Gregory A.J., Solomon G.S. (2014). McClure, D. J., Zuckerman, S. L., Kutscher, S. J., Gregory, A. J., & Solomon, G. S. (2014). Baseline neurocognitive testing in sports-related concussions: The importance of a prior night’s sleep. *American Journal of Sports Medicine*, 42(2), 472–478.	SCC	Conference Paper	50	8.3
24	Cardinali, D. P., Bortman, G. P., Liotta, G., Lloret, S. P., Albornoz, L. E., Cutrera, R. A., ... & Gallo, P. O. (2002). A multifactorial approach employing melatonin to accelerate resynchronisation of sleep–wake cycle after a 12 time-zone westerly transmeridian flight in elite soccer athletes. *Journal of Pineal Research*, 32(1), 41–46.	SCC	Article	47	2.6
25	Fullagar, H. H., Duffield, R., Skorski, S., Coutts, A. J., Julian, R., & Meyer, T. (2015). Sleep and Recovery in Team Sport: Current Sleep-Related Issues Facing Professional Team-Sport Athletes. *International Journal of Sports Physiology and Performance*, 10(8), 950–957.	MCC	Review	42	8.4

CD = citation density; SAP = single authored paper; SCC = single country collaboration; MCC = multi country collaboration; R = rank; *did not contain data about author affiliations, thus, the type of collaboration for it could not be determined. Please note type of paper as per Scopus classification.

**Table 5:** Top 10 highly productive countries for sleep research in athletes (*n* = 313).

Country	Papers (%)	Citations	CPP	h-index	SCC	MCC	SAP	Authors (min-max)	AAPP
Australia	97 (31.0)	1529	15.8	21	49	43	5	520 (1–18)	5.4 (2.8)
United States	66 (21.1)	736	11.2	15	41	19	6	333 (1–16)	5.1 (3.2)
France	25 (8.0)	297	11.9	9	13	11	1	133 (1–12)	5.3 (2.2)
United Kingdom	24 (7.7)	475	19.8	10	12	12	0	111 (2–8)	4.6 (1.7)
Germany	20 (6.4)	442	22.1	10	2	17	1	129 (1–14)	6.5 (3.5)
Qatar	18 (5.8)	134	7.4	7	0	17	1	121 (1–14)	6.7 (3.6)
Japan	16 (5.1)	39	2.4	4	14	1	1	98 (1–13)	6.1 (3.2)
Brazil	15 (4.8)	64	4.3	4	11	4	0	114 (3–13)	7.6 (2.7)
Italy	15 (4.8)	46	3.1	4	8	7	0	84 (3–12)	5.6 (2.3)
New Zealand	15 (4.8)	67	4.5	4	5	10	0	66 (2–10)	4.4 (2.3)

CPP = citations per paper; AAPP = average authors per paper; SAP = single authored paper; SCC = single country collaboration; MCC = multi-country collaboration.

**Table 6:** Top 10 highly productive institutions for sleep research in athletes (*n* = 313).

Affiliation	Country	Papers (%)	Citations	CPP	h-index
Australian Institute of Sport	Australia	47 (15.0)	760	16.2	16
Central Queensland University	Australia	21 (6.7)	287	13.7	9
University of Technology Sydney	Australia	15 (4.8)	400	26.7	9
University of Western Australia	Australia	13 (4.2)	135	10.4	6
Aspetar Orthopaedic and Sports Medicine Hospital	Qatar	13 (4.2)	91	7.0	6
University of Waikato	New Zealand	10 (3.2)	21	2.1	3
University of Queensland	Australia	9 (2.9)	48	5.3	4
Universität des Saarlandes	Germany	9 (2.9)	274	30.4	6
Universidade Federal de Sao Paulo	Brazil	8 (2.6)	44	5.5	3
Liverpool John Moores University	UK	8 (2.6)	238	29.8	5

CPP = citations per paper.

**Table 7:** Top 10 highly productive authors for sleep research in athletes (*n* = 313).

Author	Papers (%)	FAP	SAP	Citations	CPP	h-index *	Affiliation **
Halson, S.L.	25 (8.0)	3	4	431	17.2	9	Australian Catholic University, North Sydney, NSW, Australia
Sargent, C.	20 (6.4)	5	11	286	14.3	9	Central Queensland University, Adelaide, Australia
Lastella, M.	16 (5.1)	9	1	216	13.5	5	Central Queensland University, Adelaide, Australia
Roach, G.D.	16 (5.1)	1	4	221	13.8	7	Central Queensland University, Adelaide, Australia
Duffield, R.	15 (4.8)	1	1	400	26.7	9	University of Technology Sydney, Sydney, Australia
Dawson, B.	9 (2.9)	0	0	110	12.2	5	West Coast Eagles Football Club, Perth, Australia
Meyer, T.	9 (2.9)	0	6	274	30.4	6	Universität des Saarlandes, Saarbrucken, Germany
Eastwood, P.R.	8 (2.6)	0	7	74	9.3	4	University of Western Australia, Perth, Australia
Gore, C.J.	8 (2.6)	0	0	167	20.9	7	Australian Institute of Sport, Canberra, Australia
Chamari, K.	7 (2.2)	0	2	60	8.6	5	Aspetar Orthopaedic and Sports Medicine Hospital, Doha, Qatar

CPP = citations per paper; FAP = first author paper; SAP = senior author paper; * related to their papers in sleep in athletes; ** affiliations as suggested by Scopus database at the time of data analysis.

## Supplementary Materials

The following are available online at https://www.mdpi.com/2624-5175/2/2/10/s1.

## Appendix A

Search strategy used for the literature search:

TITLE (“sleep*” OR “sleep quantity” OR “sleep quality” OR “sleep-hygiene” OR “sleep disturbance” OR “nap*” OR “wak*” OR “wake”) AND TITLE (“athlet*” OR “player*” OR “sport*” OR “cyclist*” OR “football*” OR “basketball*” OR “netball*” OR “competit*” OR “team sport*” OR “individual sport*” OR “cricket*” OR “game*” OR “handball*” OR “baseball*” OR “softball*” OR “rugby” OR “cycling” OR “soccer” OR “*hockey” OR “futsal” OR “volleyball” OR “korfball” OR “lacrosse” OR “curling” OR “polo” OR “*swimming” OR “rowing” OR “mountain biking” OR “triathlon” OR “race walking” OR “canoeing” OR “diving” OR “running” OR “judo” OR “*skating” OR “ballet” OR “skiing” OR “snowboarding” OR “taekwondo” OR “archery” OR “badminton” OR “bmx” OR “bocce” OR “bowling” OR “boxing” OR “cheerleading” OR “croquet” OR “dance” OR “fencing” OR “golf” OR “gymnastics” OR “martial arts” OR “squash” OR “throw” OR “*tennis” OR “karate” OR “touch football*” OR “ice-hockey” OR “synchronised swimming” OR “swimming” OR “speed skating” OR “figure skating” OR “table tennis” OR “weight lift*” OR “roller skating” OR “motor sport*” OR “orienteering” OR “equestrian” OR “wheelchair prop” OR “water polo” OR “abseiling” OR “surfing”) AND PUBYEAR > 1965 AND (LIMIT-TO (SRCTYPE, “j”)) AND (EXCLUDE (DOCTYPE, “er”))

## Appendix B

Co-authorship analysis for countries with minimum five papers produced the network visualisation map of 15 countries in five clusters, each represented by a distinct colour. The strongest collaboration was present among Australia–Germany (link strength = 13), Australia–Qatar (link strength = 10), and Australia–New Zealand (link strength = 7).
